# Early Onset of Hypersynchronous Network Activity and Expression of a Marker of Chronic Seizures in the Tg2576 Mouse Model of Alzheimer’s Disease

**DOI:** 10.1371/journal.pone.0119910

**Published:** 2015-03-13

**Authors:** Charlotte Bezzina, Laure Verret, Cécile Juan, Jessica Remaud, Hélène Halley, Claire Rampon, Lionel Dahan

**Affiliations:** 1 Université de Toulouse; UPS; Centre de Recherches sur la Cognition Animale; 118 route de Narbonne, F-31062, Toulouse, Cedex 09, France; 2 CNRS, Centre de Recherches sur la Cognition Animale, F-31062, Toulouse, France; IGBMC/ICS, FRANCE

## Abstract

Cortical and hippocampal hypersynchrony of neuronal networks seems to be an early event in Alzheimer’s disease pathogenesis. Many mouse models of the disease also present neuronal network hypersynchrony, as evidenced by higher susceptibility to pharmacologically-induced seizures, electroencephalographic seizures accompanied by spontaneous interictal spikes and expression of markers of chronic seizures such as neuropeptide Y ectopic expression in mossy fibers. This network hypersynchrony is thought to contribute to memory deficits, but whether it precedes the onset of memory deficits or not in mouse models remains unknown. The earliest memory impairments in the Tg2576 mouse model of Alzheimer’s disease have been observed at 3 months of age. We thus assessed network hypersynchrony in Tg2576 and non-transgenic male mice at 1.5, 3 and 6 months of age. As soon as 1.5 months of age, Tg2576 mice presented higher seizure susceptibility to systemic injection of a GABA_A_ receptor antagonist. They also displayed spontaneous interictal spikes on EEG recordings. Some Tg2576 mice presented hippocampal ectopic expression of neuropeptide Y which incidence seems to increase with age among the Tg2576 population. Our data reveal that network hypersynchrony appears very early in Tg2576 mice, before any demonstrated memory impairments.

## Introduction

Alzheimer’s disease (AD) is a neurodegenerative disease characterized by several cognitive and behavioral troubles attacking particularly memory functions. Most of AD patients develop detectable symptoms after 65 year-old (sporadic cases). However, in familial forms of the disease representing less than 1% of AD cases, severe cognitive and memory deficits develop earlier, due to the presence of mutated forms of the Amyloid Precursor Protein (APP) or Presenilin (PS-1 or PS-2) genes [1,2,3,4]. Transgenic mouse models of AD, that overexpress at least one of these human mutated genes, reproduce the amyloidopathy and some anatomical and behavioral abnormalities found in AD patients [5], and thereby help to provide insights into the mechanisms underlying memory deficits.

Recently, new hypothesis have emerged about the contribution of network hypersynchrony in memory dysfunction in AD. Multiple lines of evidence point out an increased incidence of spontaneous seizures in patients with AD. In sporadic AD, an estimated 10 to 22% of patients exhibit unprovoked seizures [6,7,8]. In familial forms of AD or diseases in which APP gene is duplicated, 30 to 60% of patients develop seizures [9,10,11]. In patients diagnosed for both epilepsy and AD (or amnestic Mild Cognitively Impairment, aMCI), seizure onset preceded or coincided with the diagnosis of the disease in 83% of patients, suggesting an early onset of epilepsy in AD [12]. Using fMRI during a task involving the hippocampus, Bakker and colleagues showed that aMCI subjects displayed hippocampal hyperactivity [13]. Interestingly, in the same study, the antiepileptic drug levetiracetam reduced hippocampal hyperactivity and improved memory performances, suggesting a causal link between network hyperactivity and memory deficits [13].

Similar to human pathology, several mouse models of AD present a combination of features revealing network hypersynchrony and epilepsy. Initially, sporadic spontaneous seizures or behavioral stereotypies were fortuitously observed during behavioral phenotyping of AD transgenic lines [14,15]. Pro-convulsant agents that exacerbate neuronal excitation (kainate) or suppress inhibitory control (GABA_A_ receptor antagonist pentylenetetrazole, PTZ) trigger more severe seizures in several AD transgenic mice (TgCRND8, hAPPJ20, hAPPJ9) than in their respective non-transgenic (NTg) littermates [16,17,18,19]. Although very infrequent, spontaneous seizures in AD mice have also been objectively evidenced by using electroencephalographic (EEG) recordings [17,20,21,22]. Typically, chronic EEG recordings over very long period of time are required for observing a significant occurrence of seizures. For instance, 25% of APP_swe_xPS1_dE9_ mice present seizures when recorded for 2 weeks [20], and about 10% of hAPPJ20 mice display seizures when recorded for 24 hours [23]. However, the vast majority—if not all—of the recorded hAPPJ20 and APP_swe_xPS1_dE9_ mice show interictal spikes consisting of frequent high-voltage sharp events [17,20].

Aside from electroencephalographic markers of neuronal network hypersynchrony, neuropeptide Y (NPY) expression pattern has been proposed as a marker for chronic seizures. NPY is an inhibitory neuromodulator normally expressed by a subset of hippocampal interneurons, which can be expressed ectopically in mossy fibers after chronic seizures in animal models of epilepsy [24]. Such ectopic expression of NPY has been reported in Tg2576 [25,26,27], hAPPJ20 [17,28], and APP_swe_xPS1_dE9_ mice [20], arguing for the existence of epilepsy in these AD models. Interestingly, chronic administration of the antiepileptic drug levetiracetam suppresses epileptiform activity and improves memory performances in hAPPJ20 mice, but also normalizes the expression of NPY in the mossy fibers [28].

In AD mouse models, neuropathological processes and memory deficits progress with age. So far, the presence of network hypersynchrony has been assessed when mice were old enough to exhibit memory deficits. We thus do not know if this abnormal brain activity occurs before, concomitantly or after the onset of memory dysfunction in AD mouse models. Learning and memory deficits have been extensively described at different ages in Tg2576 mice, which express a human APP (hAPP) gene carrying the double Swedish mutation (HuAPP696swe) [29]. No memory disorder has been reported at 1.5–2 months [30,31,32], while a few studies have evidenced impairments at 3 months [30,33,34,35], and most of authors agree on clear memory deficits after 6 months [32,36,37,38,39]. Interestingly, network hypersynchrony has not been evaluated before 6 months of age in Tg2576 mice. We thus gauged network hypersynchrony of Tg2576 mice at different ages (1.5, 3 and 6 months old) by assessing their seizure susceptibility to PTZ, and evaluating their spontaneous epileptiform activity through EEG recordings and hippocampal expression pattern of NPY. We show that Tg2576 mice display aberrant network activity as early as 1.5 month-old, that is to say before the onset of memory deficits.

## Methods

### Ethics statement

All experiments were performed in strict accordance with the policies of the European Union (86/609/EEC), the French National Committee of Ethics (87/848), and the local committee's recommendations (C 31–555–11, Direction départementale de la protection des populations) for the care and use of laboratory animals. Animal facility of the CRCA is fully accredited by the French Direction of Veterinary Services (C 31–555–11, Feb 9, 2011) and animal surgery and experimentation conducted in this study were authorized by the French Direction of Veterinary Services (#31–11555521, 2002). All efforts were made to improve animals’ welfare and minimize animals’ suffering.

### Mouse Line

Experiments were performed on male mice from the transgenic line Tg2576 [15,29] from our in-house colony [25,26,40], at 1.5, 3 and 6 months of age. Tg2576 mice overexpress a double mutant form of human APP695 (Lys670-Asn, Met671-Leu [K670N, M671L]), driven by a hamster prion protein promoter. Tg2576 males were bred with C57B6/SJL F1 females (Charles River, L’Arbresle, France) and the offspring was genotyped for the hAPP transgene using DNA obtained from postweaning tail biopsies. Polymerase chain reaction products were analyzed to confirm the presence of hAPP DNA sequence in offspring. Mice were maintained on a 12 hours light/12 hours dark cycle with free access to food and water.

### PTZ injection

Seizure susceptibility was assessed by behavioral scoring of the severity of seizures induced pharmacologically (NTg *1*.*5 month-old (mo)*: n = 16, *3 mo*: n = 15 and *6 mo*: n = 16; Tg2576 *1*.*5 mo*: n = 15, *3 mo*: n = 16 and *6 mo*: n = 11). Mice received a single i.p injection of PTZ at 40 mg/kg (PTZ, Sigma Aldrich, St Louis, MO, USA). Some mice accidentally received a lower PTZ dose and thus were excluded of the study (Tg2576 *3 mo*: n = 1 and *6 mo*: n = 1). After drug administration, each mouse was placed in a new cage and its behavior was videotaped for 20 minutes. Mice were sacrificed immediately after (as described below) in order to minimize suffering. Two mice were excluded because they presented a liver hypertrophy at autopsy that might have resulted in modifications of the drug pharmacokinetics (NTg *6 mo*: n = 2).

### Seizure severity scoring

Two investigators blind to the experimental conditions quantified the maximal seizure severity during the 20 minutes recording session, according to a published scale [17]. Seizure severity scores were as follows: 0 = normal exploratory behavior, 1 = immobility, 2 = generalized spasm, tremble, or twitch, 3 = tail extension, 4 = forelimb clonus, 5 = generalized clonic activity, 6 = bouncing or running seizures, 7 = full tonic extension, 8 = death. If seizure severity was not clear-cut, an intermediate score was given. Given the ordinal nature of the seizure severity scale, we performed non-parametric statistical tests. Kruskal-Wallis test was used, followed by Dunn’s post-hoc tests to assess genotype effect in each group of age.

### Implantation of EEG electrodes

Tg2576 mice and non-transgenic littermates free from any pharmacological treatment were used for EEG recordings (NTg *1*.*5 mo*: n = 12, *3 mo*: n = 10 and *6 mo*: n = 8; Tg2576 *1*.*5 mo*: n = 10, *3 mo*: n = 13 and *6 mo*: n = 7). Mice were anesthetized with isoflurane (2%), an incision was performed on the scalp and a local anesthetic (lidocaine 5%) was applied on it. Body temperature was maintained throughout the surgery using a mouse heating pad (Ugo Basile, Gemonio, Italy). A veterinary ophthalmic gel (Ocrygel, Laboratoire TVM, Lempdes, France) was applied on the eyes to avoid dryness. Then, the skull was drilled and two silver electrodes were placed bilaterally over the parietal cortices, and one screw was positioned through the occipital bone over the cerebellum to serve as reference and ground electrode. One EMG electrode was placed in neck muscles. Electrodes were fixed to the skull with dental cement and plugged into a miniature connector (PlasticsOne, Roanoke, NC, USA). Then, lidocaine was applied on the flesh before suturing the skin. The animals were then allowed to recover for at least 1 week during which their health status was checked every day.

### EEG recordings

One to 5 days before the recording session, mice were habituated to the recording chamber which consisted of a Plexiglas chamber (21x20x25cm) with available food and water, placed in a Faraday cage. They were first placed in the recording chamber for 15 minutes without being connected to the EEG recording system through the cable and brought back to their home cage for at least 15 minutes. They were then placed again in the recording chamber and connected with the EEG cable for 2.5 hours under the supervision of an experimenter. On the recording day, mice EEG were monitored during 2.5 hours. For this purpose, they were connected with a six-channel cable (PlasticsOne) and head-staged with a home-made tension follower. This cable was connected to a multichannel commutator (PlasticsOne) that allows mice to freely move. EEG and EMG signals were amplified and band-pass filtered (for EEG: 0.3–100 Hz; for EMG: 3 Hz-20 kHz) using a AM system 3500 amplifier (A-M system, Sequim, WA, USA) and sampled at 1kHz (Power 1401 mk-II, CED, Cambridge, UK). EEG recordings were analyzed using Spike 2 V7.11 software. After EEG recordings, mice were sacrificed as described below.

### Detection of epileptiform abnormalities on EEG traces

Each digitized EEG file was screened for epileptiform activity by an investigator blind to experimental conditions. Epileptiform activity is described as the occurrence of interictal spikes, defined as sharp (2 to 50 ms) positive and/or negative deflections with amplitudes exceeding twice the baseline EEG recording [20]. Specifically, all EEG deflections that reached a two-fold baseline threshold were automatically detected using Spike 2 software. Were considered as interictal spikes only the events matching both morphological and temporal criteria (2 to 50 ms). Then, for each mouse, an average of the spike waveform was calculated and the spike duration was measured between the start of the negative deflection and the peak of the positive deflection. Epileptiform activity quantification consisted in counting the number of interictal spikes per minute during the last hour of recordings, when mice have become habituated to the recording setup. We excluded four animals because of movements artifacts on EEG traces (NTg *1*.*5 mo*: n = 2, *3 mo*: n = 1 and *6 mo*: n = 1). Statistical analysis of the frequency of interictal spikes (spikes/minute) was performed using a two-way ANOVA, followed by a Bonferroni post-hoc test.

### Tissue processing and NPY immunohistochemistry

Mice that underwent PTZ or EEG experiments were deeply anesthetized using pentobarbital and transcardially perfused with 0.9% saline solution (NTg *1*.*5 mo*: n = 20, *3 mo*: n = 25 and *6 mo*: n = 24; Tg2576 *1*.*5 mo*: n = 19, *3 mo*: n = 29 and *6 mo*: n = 18). The brains were post-fixed for 2 days in 4% paraformaldehyde and transferred into 30% sucrose in 0.1 M phosphate buffer containing 0.1% sodium azide before being cut into 30μm thick cryostat coronal sections. The sections were then stored at -20°C in a cryoprotectant solution until use. Free-floating brain sections were rinsed in phosphate-buffered saline containing 0.25% Triton X-100 (PBST). Sections were quenched 15 minutes for endogenous peroxidases with 3% H_2_O_2_ in 10% methanol/phosphate-buffered saline, then they were incubated overnight in primary antibody rabbit anti-NPY (1:5,000; Sigma Aldrich, St Louis, MO, USA) in PBST containing 0.1% sodium azide with 5% normal goat serum. The next day, the sections were incubated for 90 minutes in biotinylated goat anti-rabbit antiserum (1:250; Vector Labs, Burlingame, CA, USA) in PBST, followed by 90 minutes in avidin-biotin-peroxidase complex (1:400; ABC kit, Vector Labs) in PBST. The peroxidase immunolabeling was developed in 0.05 M Tris-HCl buffer (pH 7.6) containing 0.025% 3,3’-diaminobenzindine-4 HCl (DAB; Sigma Aldrich), 0.003% H_2_O_2_ and 0.06% nickel ammonium sulfate. The reaction was stopped by extensive rinses in PBST containing 0.1% sodium azide. The sections were mounted onto subbed slides, dehydrated through alcohols, and coverslipped.

### Qualitative assessment of NPY ectopic expression and statistical analysis

NPY ectopic expression was visually assessed by two independent observers blinded to the mouse genotype. The presence of ectopic expression was revealed by a strong immunoreactivity in the hilus and stratum lucidum regions, where mossy fibers extend their axonal processes. Twenty four out of 135 mice were excluded for the following reasons: 2 had hypertrophied liver (NTg, *6 mo*: n = 2), 13 for cryostat cutting problems (Tg2576: *1*.*5 mo*: n = 1, *3 mo*: n = 4, *6mo*: n = 3; NTg: *3 mo*: n = 2, *6mo*: n = 3), 7 for sample conservation issues (Tg2576: *1*.*5 mo*: n = 1, *3 mo*: n = 2, *6mo*: n = 3; NTg: *6mo*: n = 1), 2 mice died prior to sacrifice (Tg2576,*3 mo*: n = 2). The proportion of animals showing NPY ectopic expression was compared between non-transgenic and transgenic animals, at each age using the Fisher exact test. The proportion of Tg2576 mice showing NPY ectopic expression at different ages was compared using the Chi square test for trend.

All statistical analysis were performed using the Prism 5 software (GraphPad Software Inc., La Jolla, CA, USA).

## Results

### Tg2576 mice show higher susceptibility to pharmacologically-induced seizures from early age on

**Fig 1:**
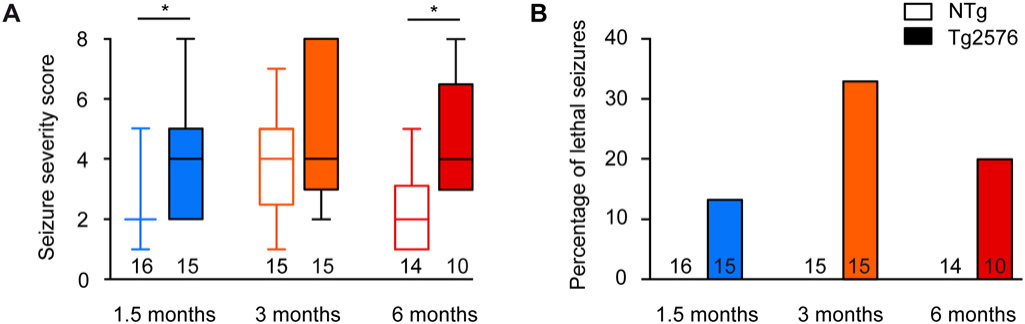
Tg2576 mice show high susceptibility to pharmacologically-induced seizures from early age on. (A) Seizure severity score of 1.5, 3 and 6 month-old Tg2576 male mice and non-transgenic (NTg) age-matched littermates. Whiskers boxes represent the interquartile distribution. Number of mice in each group is indicated below the boxes. Tg2576 mice exhibit more severe seizures than NTg at 1.5 and 6 months of age (Dunn’s tests: p<0.05 for Tg2576 vs NTg at 1.5 and 6 month-old). (B) Proportion of animals that died during PTZ-induced seizures in 1.5, 3 and 6 month-old Tg2576 and NTg mice. Note that only transgenic animals exhibit lethal seizures. Numbers over the horizontal axis indicate the number of mice used in each experimental group.

First, we determined seizure susceptibility of Tg2576 mice and their NTg littermates at ages of 1.5, 3 and 6 months. Injection of the GABA_A_ receptor antagonist PTZ, at the dose of 40 mg/kg, induced more severe seizures in Tg2576 mice than in NTg littermates at 1.5 and 6 months of age (Fig. 1A, Kruskal-Wallis: p<0.0001, Dunn’s post hoc tests: p<0.05 for NTg *vs* Tg2576 at 1.5 and 6 month-old). At 3 months of age, the difference in seizure severity between NTg and Tg2576 mice did not reach statistical significance as NTg mice showed higher seizure susceptibility than at 1.5 months of age (Dunn’s post hoc test: p<0.01; Fig. 1A). Some Tg2576 animals showed lethal seizures, whilst this was never observed among NTg animals (Fig. 1B). In summary, as early as 1.5 months of age, Tg2576 mice exhibit a higher susceptibility to pharmacological seizures than NTg littermates.

### Tg2576 mice exhibit spontaneous epileptiform activity as young as 1.5 months of age

**Fig 2:**
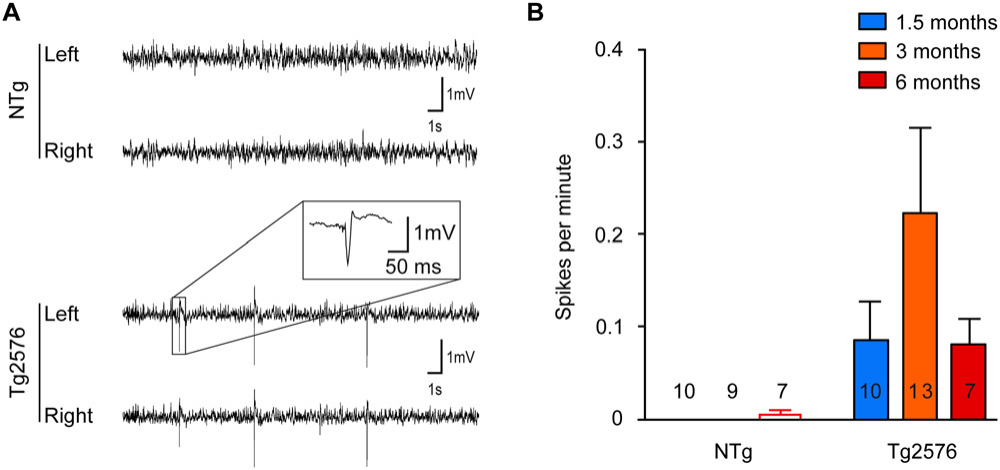
Tg2576 mice exhibit spontaneous epileptiform activity as young as 1.5 months of age. (A) Representative electroencephalographic (EEG) traces from non-transgenic (NTg) (top) and Tg2576 (bottom) mice from left and right parietal cortices. Note that only transgenic animals displayed sharp, high-voltage spikes that characterize epileptiform activity (inset). (B) Quantitative analysis of the frequency of interictal spikes (mean ± SEM). Two-way ANOVA shows a significant genotype effect (p = 0.013) but no age effect (p = 0.4091) and no interaction (p = 0.3865). Numbers over the horizontal axis indicate the number of mice used in each experimental group.

Then, we examined to which extent such susceptibility might be associated to spontaneous electroencephalographic (EEG) abnormalities in Tg2576 mice. We recorded cortical EEG in Tg2576 and NTg mice at 1.5, 3 and 6 months of age. During the recording session, we did not observe any electroencephalographic seizures in NTg nor in Tg2576 mice. Since spontaneous seizures are relatively rare events in other mouse models of Alzheimer’s disease [20, 23] and given our recording time window, this was highly expected. Nevertheless, interictal spikes were observed in most of Tg2576 mice (63% of mice, regardless of their age). These events lasted 21.2 ± 2.6 ms and displayed the characteristic shape of interictal spikes [20] (Fig. 2A). In NTg mice, only 1 mouse out of 26 displayed spikes at a very low frequency (0.01 spike/minute) (Fig. 2B). Spike frequency was significantly influenced by the genotype but not by the age of the animals (Fig. 2B, two-way ANOVA; transgene effect: p = 0.013; age effect: p = 0.4091; interaction: p = 0.3865). These results clearly show that spontaneous epileptiform activity is already present in Tg2576 mice as early as 1.5 months of age.

### Ectopic expression of NPY in the mossy fibers of young Tg2576 mice

**Fig 3:**
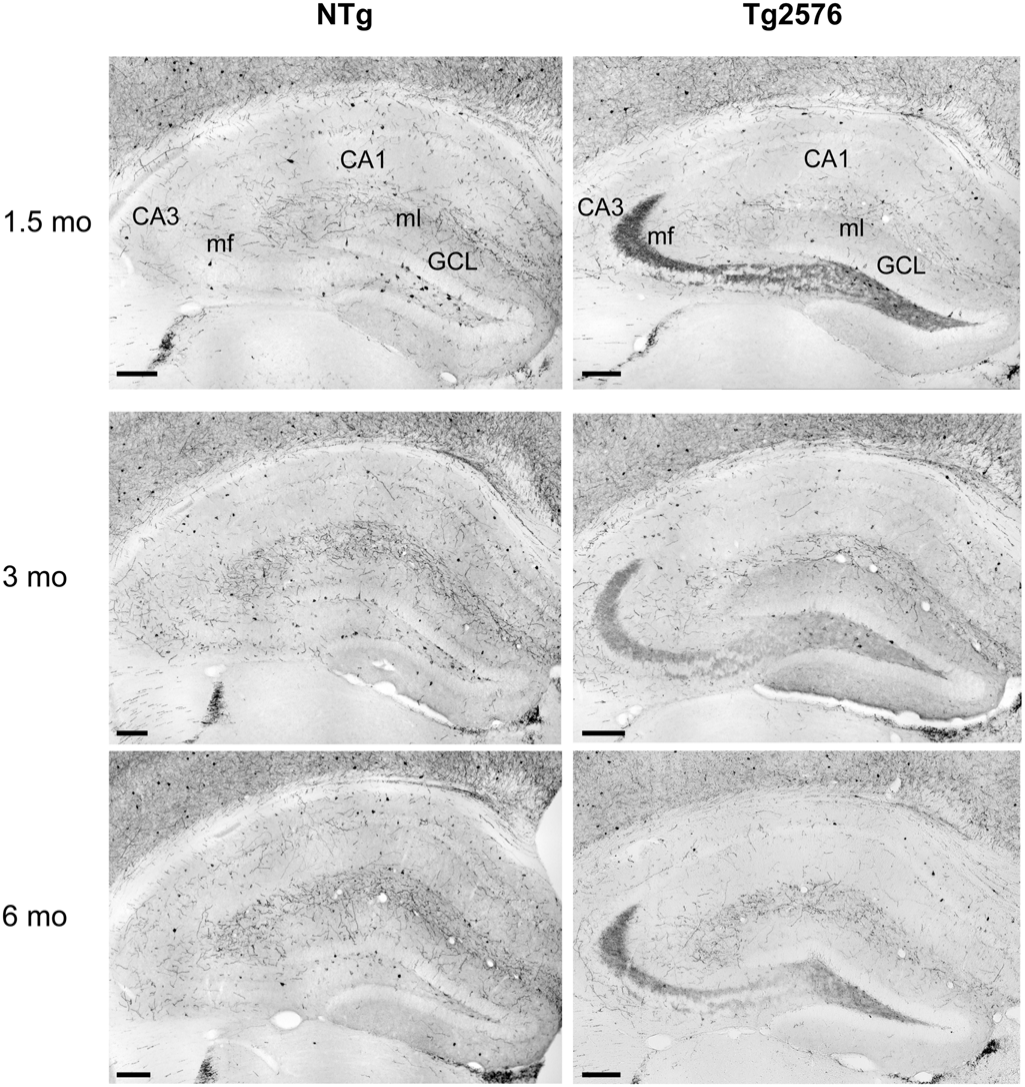
Ectopic expression of NPY in the mossy fibers of young Tg2576 mice. Photographs of the dorsal hippocampus immunostained for NPY in 1.5, 3 and 6 month-old non-transgenic (NTg) and Tg2576 mice. Left: In NTg animals, NPY staining is visible in the soma of hilar interneurons. Their axons display a faint staining visible in the molecular layer (ml), where these axons form synapses onto the dendrites of granular cells. Right: Typical ectopic NPY expression in the mossy fibers (in *hilus* and *stratum lucidum*) of a Tg2576 mouse. CA1 and 3, *Cornu Ammonis* 1 and 3, GCL: Granular Cell Layer, mf: mossy fibers, ml: molecular layer. Scale bar: 200 μm.

To assess the occurrence of chronic seizures in Tg2576 mice, we looked for NPY ectopic expression in mossy fibers of the dentate gyrus by using NPY immunohistochemistry (Fig. 3). While never observed in the mossy fibers of NTg animals, NPY ectopic expression was found in a significant proportion of Tg2576 animals (Table 1, Chi square test for genotypes, regardless of the age: p = 0.0002). NPY ectopic expression was observed as young as 1.5 month of age. The proportion of mice showing NPY ectopic expression was significantly higher in Tg2576 than in NTg littermates at 3 and 6 months of age but not at 1.5 months of age (Table 1). Although not statistically significant in this sample, the proportion of Tg2576 mice showing NPY ectopic expression seemed to increase with age (Table 1, Chi square test for trend: p = 0.16). These results suggest that chronic seizures occur at very early stages in the course of the disease, and that their incidence likely increases with age among the Tg2576 population.

**Table 1:** Proportion of mice with NPY ectopic expression among Tg2576 mice and NTg littermates.

Age (months)	NTg	Tg2576	Fisher exact test for NTg vs Tg2576
1.5	0/20 (0%)	2/17 (11.7%)	p = 0.204
3	0/23 (0%)	4/21 (19.0%)	p = 0.044
6	0/18 (0%)	4/12 (33.3%)	p = 0.018

Data represent the number of animals with NPY ectopic expression in mossy fibers compared to the total number of animals in each group. Note that only Tg2576 mice present aberrant NPY expression.

## Discussion

This study reports that at 1.5, 3 and 6 months of age, Tg2576 mice exhibit high susceptibility to pharmacologically-induced seizures, EEG epileptiform activity and NPY ectopic expression in mossy fibers, this later marker of chronic seizures showing an increased incidence with age. Our work provides the first evidence that such network dysfunction precedes the onset of memory deficits.

A few studies have previously assessed network hypersynchrony in the Tg2576 mouse line. To date, there is no data concerning the sensitivity of Tg2576 mice to convulsive agents, except a study showing an increased sensitivity to PTZ in Tg2576 mice bred on a pure C57bl/6 background, which exhibit an unusually high mortality rate (40% died before they reached 2 months of age) and thus cannot be considered as a typical Tg2576 line [41]. In the original Tg2576 line, EEG abnormalities were reported at 5–7 months of age but were not clearly described. The authors related longer durations of “higher frequency brain activity” (6 to 10 Hz), which they interpret as an increased synchrony, but did not report any obvious spike or seizure [27]. Thus, our data constitute the first clear demonstration of higher sensitivity to convulsive agents and occurrence of spontaneous interictal spikes in this mouse line. Ectopic expression of NPY in the mossy fibers has often been associated with epileptic activity in the hippocampus or with the occurrence of a generalized seizure in the days before sacrifice and can thus be used as a marker for chronic seizures [24,42]. Ectopic expression of NPY in the Tg2576 mice was evidenced at 5–7 months of age [27] and previous data from our group showed that the proportion of mice presenting this marker of chronic seizures increases with age (11% at 3 months, 60% at 18 months) [25]. Our present data confirm this observation and further point out that this marker can already be observed as soon as 1.5 months of age. Altogether, our data strongly suggest that Tg2576 mice present a precocious epileptic phenotype. Interestingly, previous work showed that Tg2576 exhibit normal memory performances at 1.5–2 months of age [30,31], memory deficits appearing in this mouse line between 3 and 6 months of age [30,31,32]. Thus, network hypersynchrony and expression of markers of chronic seizures occur before the onset of memory deficits in Tg2576 mice.

Network hypersynchrony and hyperexcitability have been described in other mouse models of AD. In the TgCRND8 line, seizure susceptibility to PTZ was evidenced at 6–8 weeks of age [16] when these mice already present memory deficits [43]. In APP_*Swe*_xPS1_dE9_ mice, electroencephalographic seizures and epileptiform activity were found at 3–4 months of age [20], corresponding to the earliest description of memory deficits in this mouse line [44]. In hAPPJ20 mice, susceptibility to pharmacological seizures, spontaneous seizures, interictal spikes and NPY ectopic expression were described at 4–7 months of age [17,18,23,28], when mice already exhibit memory deficits from 2–3 months of age [45]. Thus, although it has never been assessed, network hypersynchrony might also happen before the onset of memory deficits in these models. Interestingly, this is also supported by data from human cases suggesting that epileptic events can precede the onset of memory impairments in AD and aMCI [12].

Several hypotheses can be proposed to explain the precocity of epileptic activity in Tg2576 mice. The Tg2576 mouse model expresses a mutated form of hAPP inducing an excessive production of Aβ1–42 peptide and its accumulation into amyloid plaques [46]. In the APP_swe_xPS1_G384A_ mice model, neurons located in the vicinity of amyloid plaques were reported to be hyperactive [47]. However, Tg2576 mice are completely devoid of amyloid plaques at 1.5 months of age [46], ruling out the possibility that plaques could be responsible for the early onset of network hyperactivity nor hypersynchrony in these mice. Nevertheless, the brains of new born Tg2576 mice, but not of NTg mice, already contain soluble Aβ1–42 [48]. At the cellular level, soluble Aβ1–42 species were suggested to play a role in neuronal hyperexcitability of AD mice. For instance, bath application of soluble Aβ decreases depolarization threshold and thus increases excitability of pyramidal cortical neurons or granule cells of the dentate gyrus [20,21] and intra-hippocampal injection of Aβ oligomers was found to increase population spikes evoked by perforant path stimulation in rats [49]. However, the molecular and cellular mechanisms underlying the effects of Aβ on neuronal hyperexcitability are still unclear. Recently, Lee et al. reported that Aβ disturb mitochondrial function in 1.5 month-old Tg2576 mice, leading to a slower decay of Ca^2+^ transients in granule cells of the dentate gyrus [50]. Further work is needed to determine if this increase in intracellular Ca^2+^ signal in granule cells could participate to network hypersynchrony in 1.5 month-old Tg2576 mice.

Interestingly, the role of Aβ in network hypersynchrony has been recently challenged. The APP_Swe/Lon_xPS1_M146V_ mouse line, which produces high amounts of Aβ, does not exhibit any epileptiform activity even as late as 23 months of age [22]. Born and colleagues proposed that mutant APP itself could be involved in network hypersynchrony. Indeed, APP is a transmembrane protein cleaved by secretases as the β-site APP cleaving enzyme 1 (BACE1). In transgenic models of AD that overexpress mutant APP, excessive levels of this full-length APP may hijack a significant portion of BACE1, thus reducing its ability to process other substrates such as sodium channels subunits. Indeed, BACE1 cleaves the Navβ2 subunit of Nav1.1 channels, which regulates the expression of the functional α-subunits of these channels [51] that control excitability of parvalbumin-expressing interneurons [52]. Reduced levels of Nav1.1 channels were reported in association with impaired function of interneurons leading to network hypersynchrony and memory deficits in hAPPJ20 [23] and 5- to 7-month-old Tg2576 mice [27]. To which extent high levels of mutant APP reduce Nav1.1 levels in parvalbumin-expressing interneurons in young Tg2576 mice remains to be determined.

Recent studies clearly suggest a role of network hypersynchrony in memory deficits. In aMCI subjects, the antiepileptic drug levetiracetam improves memory performances [13]. In hAPPJ20 mice, one month of chronic levetiracetam treatment suppresses epileptiform activity, normalizes hippocampal NPY expression and improves memory performances [28]. The beneficial effect of levetiracetam on memory performances in hAPPJ20 mice may result from the suppression of epileptiform activity or the normalization of NPY expression in the hippocampus or both. In a rat model of epilepsy and epileptic patients, hippocampal interictal spikes occurring during memory retrieval impair memory performances [53,54]. Here we report epileptiform activity in 1.5 month- old Tg2576 mice, an age when these mice present normal memory performances [30,31]. Thus, memory deficits may not result from hypersynchrony and epileptiform activity themselves, but rather from the consequences of their chronicity. Chronic hypersynchrony triggers seizures which incidence increases with age as reported in the APP_swe_xPS1_dE9_ model of AD (15% of mice had seizures at 3 months *vs* 50% at 4 months of age) [20]. Consistent with these results, the present study and previous data from our laboratory [25] report an age-related increase in the incidence of NPY ectopic expression which is significant from NTg mice at 3 months and clearly rises around 6 months of age. This parallels the progression of memory deficits, which begin at 3 months, and progressively worsen with age [30,31,32]. Expression of NPY in granule cells decreases glutamatergic synaptic transmission [55,56]. If it prevents a spread of neuronal overexcitation in a context of chronic seizures, it could also impair hippocampal function required for learning and memory processes. Thus, early network hypersynchrony induces seizures which in turn trigger neuroadaptations in the hippocampus, including NPY ectopic expression, which might cause a progressive degradation of hippocampal function explaining the age-dependent decline in memory performances in AD mice [57].

Network hypersynchrony could also cause memory impairment by altering neurogenesis. Adult hippocampal neurogenesis, a process by which new granule cells of the dentate gyrus are generated throughout life, contributes to learning and memory [58,59]. In AD mice, altered neurogenesis seems to be an early event in the course of the disease [25,60]. We recently described an impairment of adult hippocampal neurogenesis at 3 months of age in Tg2576 mice [25]. At this specific age, adult-generated neurons of Tg2576 mice exhibit impaired neuronal maturation and reduced dendritic spine density and dendritic length. Similar observations were made in hAPPJ20 mice [61]. In Tg2576 mice and hAPPJ20, altered neurogenesis has been described at the onset of memory deficits at 3 months of age, but it cannot be excluded that such alterations may begin earlier. In hAPPJ20 mice, Sun and colleagues showed that the excitation/inhibition imbalance contributes to adult neurogenesis impairments [61]. Whether alteration of hippocampal neurogenesis is present before memory deficits and whether it relates to hypersynchrony in Tg2576 mice remains to be established.

## Conclusion

Here we evidence network hypersynchrony before the onset of memory deficits in Tg2576 mice and an age-related increase of the incidence of ectopic NPY in the mossy fibers of Tg2576 mice revealing an increasing frequency of seizures with age. This early network dysfunction could initiate progressive modifications of hippocampal network leading *in fine* to overt memory dysfunction. In human, network hypersynchrony would thus potentially represent an early diagnosis marker to predict memory decline. However, extrapolations of these findings to sporadic forms of the disease still remain to be investigated.
